# Diagnosing hypertension in primary care: a retrospective cohort study to investigate the importance of night-time blood pressure assessment

**DOI:** 10.3399/BJGP.2022.0160

**Published:** 2022-11-01

**Authors:** Laura C Armitage, Shaun Davidson, Adam Mahdi, Mirae Harford, Richard McManus, Andrew Farmer, Peter Watkinson, Lionel Tarassenko

**Affiliations:** Nuffield Department of Primary Care Health Sciences, University of Oxford, Oxford.; Intitute of Biomedical Engineering, University of Oxford, Oxford.; Oxford Internet Institute, University of Oxford, Oxford.; Nuffield Department of Clinical Neurosciences, University of Oxford, Oxford.; Nuffield Department of Primary Care Health Sciences, University of Oxford, Oxford.; Nuffield Department of Primary Care Health Sciences, University of Oxford, Oxford.; Nuffield Department of Clinical Neurosciences, University of Oxford, Oxford.; Intitute of Biomedical Engineering, University of Oxford, Oxford.

**Keywords:** ambulatory blood-pressure monitoring, blood pressure monitoring, cardiovascular disease, hypertension

## Abstract

**Background:**

Ambulatory blood-pressure monitoring (ABPM) has become less frequent in primary care since the COVID-19 pandemic, with home blood-pressure monitoring (HBPM) often the preferred alternative; however, HBPM cannot measure night-time blood pressure (BP), and patients whose night-time BP does not dip, or rises (reverse dipping), have poorer cardiovascular outcomes.

**Aim:**

To investigate the importance of measuring night-time BP when assessing individuals for hypertension.

**Design and setting:**

Retrospective cohort study of two patient populations — namely, hospital patients admitted to four UK acute hospitals located in Oxfordshire, and participants of the BP in different ethnic groups (BP-Eth) study, who were recruited from 28 UK general practices in the West Midlands.

**Method:**

Using BP data collected for the two cohorts, three systolic BP phenotypes (dipper, non-dipper, and reverse dipper) were studied.

**Results:**

Among the hospital cohort, 48.9% ( *n* = 10 610/21 716) patients were ‘reverse dippers’, with an average day–night systolic BP difference of +8.0 mmHg. Among the community (BP-Eth) cohort, 10.8% ( *n* = 63/585) of patients were reverse dippers, with an average day–night systolic BP difference of +8.5 mmHg. Non-dipper and reverse-dipper phenotypes both had lower daytime systolic BP and higher night-time systolic BP than the dipper phenotype. Average daytime systolic BP was lowest in the reverse-dipping phenotype (this was 6.5 mmHg and 6.8 mmHg lower than for the dipper phenotype in the hospital and community cohorts, respectively), thereby placing them at risk of undiagnosed, or masked, hypertension.

**Conclusion:**

Not measuring night-time BP puts reverse-dippers (those with a BP rise at night-time) at risk of failure to identify hypertension. As a result of this study, it is recommended that GPs should offer ABPM to all patients aged ≥60 years as a minimum when assessing for hypertension.

## INTRODUCTION

The circadian pattern of blood pressure (BP) and its pathophysiological impact have been studied extensively over the last few decades. The usual circadian pattern, which depends primarily on the sleep–wake cycle, consists of a decrease in BP during sleep (described as a ‘dipper pattern’), usually ascribed to a reduction in sympathetic tone and an increase in vagal activity;^1^ this is then followed by a morning increase and minor oscillations during the day.

The phenotypic classification of BP, dividing people into ‘dippers’ and ‘non-dippers’ (minimal night-time BP decrease compared with daytime BP), has been used since 1988.^2^^,^^3^ Mention of a third phenotype (‘reverse dipper’) appeared in the literature in the 1990s.^4^^,^^5^ This characterises individuals whose average night-time BP is greater than their average daytime BP.

A study of 7458 people in multiple countries^4^ showed that night-time BP, adjusted for daytime BP, was a predictor of total, cardiovascular, and non-cardiovascular mortality. Reverse dippers were found to be older, were more likely to come from South America or Asia, and were at higher risk of death. A European study found that the night–day systolic BP ratio independently predicted all-cause mortality and cardiovascular events, which persisted after additional adjustment for 24-hour systolic BP.^5^ Two very recent articles reach similar conclusions: a population-based study in which night-time BP was adjusted for daytime BP showed that night-time BP was a stronger prognostic predictor than daytime BP;^6^ and, in a review studying the prevention, detection, and management of high BP, Carey and Whelton^7^ highlighted Kario *et al*’s Japan Ambulatory Blood Pressure Monitoring Prospective (JAMP) study^8^ — this investigated the association between night-time BP patterns and cardiovascular events and, again, reverse dipping was found to be statistically significantly associated with higher cardiovascular disease (CVD) risk.

The prevalence of reverse dipping has been reported to be between 3% and 39%,^1^ depending on study setting and participant characteristics, especially with respect to comorbidities of interest (for example, sleep apnoea syndrome, diabetes mellitus, and known essential hypertension).

The authors recently published a retrospective analysis of 1.7 million BP measurements for hospital patients, which showed that reverse dipping was the dominant phenotype in this patient group.^9^ The analysis of nocturnal systolic BP presented in this current article is timely and important, given that formal assessment of BP via ambulatory BP monitoring (ABPM) in primary care has become less frequent since the COVID-19 pandemic, due to challenges in accessing and delivering health care. Home BP monitoring (HBPM) has provided a partial solution to these challenges,^10^ and NHS England and NHS Improvement are now distributing home BP monitors to patients to record their daytime BP as part of the Blood Pressure @home programme.^11^

**Table:** How this fits in

Since the 1990s, the phenotypic classification of 24-hour blood pressure (BP) has divided the population into ‘dippers’, ‘non-dippers’ (minimal night-time BP decrease compared with daytime BP), and ‘reverse dippers’ (night-time BP increases compared with daytime BP). There is an established body of research demonstrating that reverse dippers are at higher risk of death and that the night–day systolic BP ratio is an independent predictor of all-cause mortality and cardiovascular events. Current UK guidelines suggest clinicians should diagnose hypertension based solely on daytime BP measurements. This study revealed that a marked proportion of the cohort was reverse dippers; together with the established clinical research that has demonstrated worse cardiovascular outcomes for such patients, this highlights the need for 24-hour ambulatory BP assessments to detect and diagnose those with nocturnal hypertension, non-dipping, or reverse-dipping BP phenotypes.

In this article, the authors:

- estimate the relative prevalence of the three systolic BP phenotypes (dipper, non-dipper, and reverse dipper) in two patient cohorts of the same age group;
- investigate the association of the reverse-dipping phenotype with average daytime systolic BP; and
- discuss the implications of this association on screening for hypertension using daytime measurements in general practice.

## METHOD

### Datasets

#### Hospital BP dataset

BP measurements were collected from patients admitted to four acute hospitals in Oxford University Hospitals NHS Foundation Trust, UK, between March 2014 and April 2018. The systolic BP of patients aged ≥18 years from all wards, excluding maternity and intensive care units, was analysed. Included patients were all those with at least three recorded BP measurements, at least one of which was recorded during night-time, at least one of which was recorded during daytime, and two of which were at least 24 hours apart. This methodology has been described in two earlier studies.^9^^,^^12^

Using recorded codes from the International Classification of Diseases, 10th revision, for the eligible patients, the prevalence of cardiometabolic comorbidity (hypercholesterolaemia, coronary heart disease, coronary artery bypass graft, heart failure, transient ischaemic attack, peripheral vascular disease, atrial fibrillation, chronic kidney disease, and diabetes) among the cohort were investigated.

#### Community ABPM dataset

The BP in different ethnic groups (BP-Eth) study was an observational study conducted between June 2010 and December 2012, with patients registered at one of 28 general practices in the UK.^13^^,^^14^ Patients were aged 40–75 years with, and without, diagnosed hypertension. The BP-Eth protocol involved comparing 24-hour ABPM and GP-clinic BP measurements to investigate the association between ‘white coat’ hypertension and ethnicity. BP was measured every 30 min during the day and hourly during the night using Spacelabs 90217-1Q monitors.^14^

Analyses of the study presented here were restricted to those patients who had at least 50% of daytime (nine out of 18) and night-time (four out of eight) measurements available. The prevalence of the same cardiometabolic comorbidities searched for among the hospital BP dataset was investigated. To enable comparisons between the two datasets, analysis of the in-patient cohort was limited to patients aged 40–75 years.

### 24-hour BP profile analysis

To derive the 24-hour systolic BP profiles for the two datasets, the 24 hours from midnight to 23:59 were divided into 1-hour bands, and defined as follows:

- night-time period — from the start of the 23:00–23:59 1-hour band until the end of the 06:00–06:59 1-hour band; or
- daytime period — from the start of the 09:00–09:59 1-hour band until the end of the 17:00–17:59 1-hour band. This is the time period during which daytime BP measurements are most likely to be made in general practice.

The selection of these night-time and daytime periods creates a 2-hour gap between the end of night-time and the start of daytime, such that data from the period during which there is greatest uncertainty as to whether a BP measurement belongs to the night or day are omitted. Similarly, the night-time period is not deemed to start until 23:00 to ensure that the BP measurements used to compute night-time averages are most likely to belong to the sleep part of the sleep–wake cycle. With this clear separation between night-time and daytime periods, the authors aimed to establish whether the extra information available from night-time measurements obtained through ABPM justifies its extra cost and difficulty, compared with standard daytime measurements taken in clinic.

For each patient, BP data were assigned to one of the 24 1-hour bands and the average systolic BP for each 1-hour band was computed from all data in that band. For patients in the hospital dataset, this meant averaging data for each band from different days; each patient, therefore, contributed one 24-hour profile, regardless of their length of hospital admission. For the ABPM dataset, two measurements taken 30 min apart were averaged to derive the BP value in each daytime 1-hour band.

For both datasets, the systolic BP values in each 1-hour band were used to compute the night-time and daytime average values for that patient, enabling their 24-hour systolic BP profile to be assigned to one of the following phenotypes:

- dipper — night-time average systolic BP <90% of daytime average systolic BP;
- non-dipper — night-time average systolic BP ≥90% and <100% of daytime average systolic BP; or
- reverse-dipper — night-time average systolic BP ≥100% of daytime average systolic BP.

In this study, the ‘extreme reverse-dipper’ phenotype was introduced as a subtype of the reverse dipper; it was defined as a night-time average systolic BP of ≥110% of daytime average systolic BP.

Twenty-four-hour systolic BP profiles characterising each phenotype for each of the two datasets were obtained by aggregating the 24-hour profiles for all patients in the dataset with that phenotype.

Sex differences in BP trajectories over the life course have recently been highlighted,^15^ and so data analyses were repeated separately for males and females.

## RESULTS

### Hospital dataset

In total, 21 716 patients aged 40–75 years met the eligibility criteria during the study period. Of these, 7220 (33.2%) had a preceding diagnosis of hypertension. The mean age was 60.6 years (standard deviation [SD] 9.9 years) and 51.1% were male. In total, 48.9% of hospital patients aged 40–75 years were reverse dippers. Table 1 details the average number of BP measurements available for the 21 716 participants eligible for inclusion in the analysis.

**Table 1.** Average number of BP measurements, per participant, contributing to the analysis of hospital 24-hour BP phenotypes

**Category**	**Whole cohort**	**Males**	**Females**
Patients, *n* (%)	21 716 (100.0)	11 111 (51.1)	10 628 (48.9)
Number of measurements, mean (SD)	39.6 (47.3)	40.8 (45.8)	38.3 (48.9)
Number of measurements, median (IQR)	26 (31)	27 (32)	25 (29)
Mean 24-hour systolic BP (SD)	126.3 (15.4)	127.5 (15.0)	125.1 (15.7)
Mean 24-hour systolic BP, median (IQR)	125.1 (20.3)	126.3 (19.6)	123.6 (20.8)

*BP = blood pressure. IQR = interquartile range. SD = standard deviation.*

**Figure 1.**
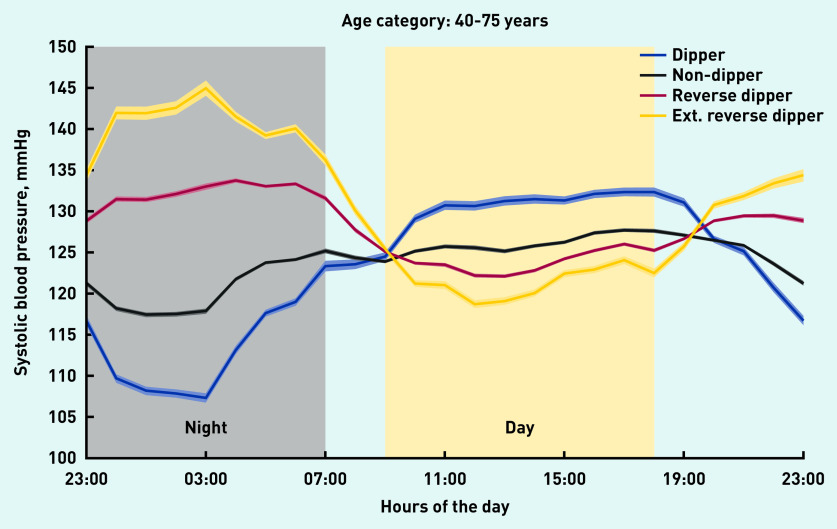
*24-hour systolic blood pressure profiles for each phenotype for hospital patients aged 40–75 years. The width of each coloured line is proportional to the variance of the data for that data point (one data point for each 1-hour bin). Ext = extreme reverse dippers.*

The average daytime systolic BP of dippers was higher than that of non-dippers and reverse dippers (Table 2 and Figure 1). For reverse dippers, the difference between average daytime and night-time systolic BP was +8.0 mmHg (Table 2). Extreme reverse dippers (a subset of reverse dippers, representing 11.9% of the 40–75-year age group) had the lowest daytime systolic BP average (122.1 mmHg), but their night-time average systolic BP was 17.3 mmHg higher at 139.4 mmHg. No evidence of a difference between males and females was found.

**Table 2.** Relative prevalence, average systolic BP, and prevalence of cardiometabolic comorbidity in hospital patients aged 40–75 years, per phenotype

**Measure**	**Phenotype**			

	**Dipper**	**Non-dipper**	**Reverse dipper**	**Extreme reverse dipper^a^**
**Whole cohort, *n*= 21 716**				
Proportion in each phenotype, *n* (%)	2290 (10.5)	8816 (40.6)	10 610 (48.9)	2590 (11.9)
Daytime systolic BP, mmHg	130.6	126.2	124.1	122.1
Night-time systolic BP, mmHg	114.1	121.5	132.1	139.4
Day–night difference, mmHg	−16.5	−4.7	+8.0	+17.3
24-hour mean systolic BP, mmHg	124.8	125.0	127.7	129.2
Prevalence of cardiometabolic comorbidity, *n* (%)	434 (19.0)	1805 (20.5)	3050 (28.7)	876 (33.8)

**Males, *n*= 11 096**				
Proportion in each phenotype, *n* (%)	1050 (9.5)	4486 (40.4)	5560 (50.1)	1332 (12.0)
Daytime systolic BP, mmHg	132.5	127.6	124.7	122.1
Night-time systolic BP, mmHg	115.9	123.1	132.8	139.6
Day–night difference, mmHg	−16.6	−4.5	+8.1	+17.5
24-hour mean systolic BP, mmHg	126.6	126.6	128.4	129.3
Prevalence of cardiometabolic comorbidity, *n* (%)	254 (24.2)	1091 (24.3)	1918 (34.5)	534 (40.1)

**Females, *n*= 10 720**				
Proportion in each phenotype, %	1240 (11.6)	4430 (41.3)	5050 (47.1)	1258 (11.7)
Daytime systolic BP, mmHg	129.0	124.6	123.5	122.0
Night-time systolic BP, mmHg	112.6	119.8	131.2	139.3
Day–night difference, mmHg	−16.4	−4.8	+7.7	+17.3
24-hour mean systolic BP, mmHg	123.2	123.4	127.0	129.1
Prevalence of cardiometabolic comorbidity, *n* (%)	180 (14.5)	714 (16.1)	1132 (22.4)	342 (27.2)

a *Extreme reverse dippers are a sub-set of the reverse-dipper phenotype. BP = blood pressure.*

Table 2 also details the prevalence of cardiometabolic comorbidity among hospital patients for each of the four systolic BP phenotypes, to help consider the cardiovascular risk of patients in each of these groups.

### Community ABPM dataset

In total, 770 primary care patients contributed ABPM data to this analysis; of those, 481 (62.5%) had a preceding diagnosis of hypertension. The mean age was 58.6 years (SD 9.6 years) and 48.6% were male. Table 3 details the average number of BP measurements available for the 585 participants eligible for inclusion in the analysis.

**Table 3.** Average number of BP measurements, per participant, contributing to the analysis of community 24-hour BP phenotypes

**Category**	**Whole cohort**	**Male**	**Female**
Patients, *n* (%)	585 (100.0)	288 (49.2)	297 (50.8)
Mean number of measurements, *n* (SD)	26.9 (4.2)	27.2 (4.0)	26.5 (4.3)
Median number of measurements, *n* (IQR)	28 (6)	28 (5)	27 (6)
Mean 24-hour systolic BP (SD)	129.5 (14.3)	130.4 (13.2)	128.6 (15.3)
Mean 24-hour systolic BP, median (IQR)	128.6 (17.2)	129.6 (14.4)	126.7 (18.7)

*BP = blood pressure. IQR = interquartile range. SD = standard deviation.*

**Figure 2.**
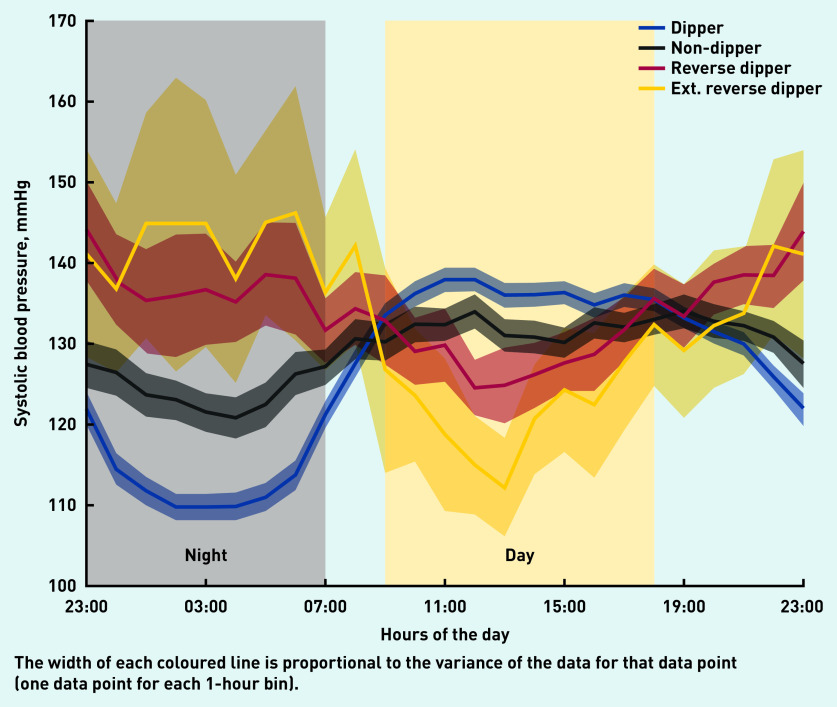
*24-hour systolic blood pressure profiles for each phenotype for community patients. Ext = extreme reverse dippers.*

Table 4 shows the numbers and percentages of the 585 patients in the ABPM dataset associated with the four systolic BP phenotypes, alongside their average daytime and night-time systolic BPs. The prevalence of reverse dipping was 10.8% in the community cohort for patients aged 40–75 years (Table 4); this demonstrates that this phenotype does exist in the community, as shown by the 24-hour systolic BP plots for the four phenotypes in Figure 2. Figure 2 also shows that dippers had a higher daytime average systolic BP than non-dippers and reverse dippers. For reverse dippers and extreme reverse dippers, the average night-time systolic BP was 8.5 mmHg and 18.8 mmHg above the average daytime systolic BP, respectively (Table 4).

**Table 4.** Relative prevalence, systolic BP measurement, and prevalence of cardiometabolic comorbidity in community patients, per phenotype

**Measure**	**Phenotype**			

	**Dipper**	**Non-dipper**	**Reverse dipper**	**Extreme reverse dipper^a^**
**Whole cohort, *n*= 585**				
Proportion in each phenotype, *n* (%)	333 (56.9)	189 (32.3)	63 (10.8)	18 (3.1)
Daytime systolic BP, mmHg	136.0	131.6	129.2	122.6
Night-time systolic BP, mmHg	112.8	123.9	137.7	141.4
Day–night difference, mmHg	−23.2	−7.7	+8.5	+18.8
24-hour mean systolic BP, mmHg	128.6	129.8	133.3	130.5
Prevalence of cardiometabolic comorbidity, *n* (%)	159 (47.7)	110 (58.2)	40 (63.5)	12 (66.7)

**Males, *n*= 288**				
Proportion in each phenotype, *n* (%)	163 (56.6)	91 (31.6)	34 (11.8)	12 (4.2)
Daytime systolic BP, mmHg	138.1	131.3	129.3	121.7
Night-time systolic BP, mmHg	114.0	123.4	137.6	138.5
Day–night difference, mmHg	−24.1	−7.9	+8.3	+16.8
24-hour mean systolic BP, mmHg	130.5	129.3	132.9	128.2
Prevalence of cardiometabolic comorbidity, *n* (%)	89 (54.6)	55 (60.4)	22 (64.7)	7 (58.3)

**Females, *n*= 297**				
Proportion in each phenotype, *n* (%)	170 (57.2)	98 (33.0)	29 (9.8)	6 (2.0)
Daytime systolic BP, mmHg	134.1	131.9	129.2	124.3
Night-time systolic BP, mmHg	111.6	124.4	137.7	147.3
Day–night difference, mmHg	−22.5	−7.5	+8.5	+23.0
24-hour mean systolic BP, mmHg	126.8	130.2	133.9	135.1
Prevalence of cardiometabolic comorbidity, *n* (%)	70 (41.2)	55 (56.1)	18 (62.1)	5 (83.3)

a *Extreme reverse dippers are a sub-set of the reverse-dipper phenotype. BP = blood pressure.*

## DISCUSSION

### Summary

Analysis of the hospital dataset showed that 48.9% of hospital patients aged 40–75 years were reverse dippers. Evidence of reverse dipping was also present in 10.8% of subjects in a smaller community dataset of ABPM measurements. Participants in the hospital cohort had a median of 26 BP measurements available for assessment of their 24-hour systolic BP phenotype; participants in the community ABPM cohort had a median of 28 measurements. The interquartile range varied markedly between the two cohorts: 31 for the hospital cohort and six for the community cohort.

The systolic BP of dippers was shown to decrease during the night (negative half-cycle), before increasing from the early morning onwards (positive half-cycle). With reverse dippers, the order of the two half-cycles in the systolic BP profile was reversed: the night-time rise (positive half-cycle) was followed by the negative half-cycle during the day; with this phenotype, systolic BP was lowest during the daytime period. As with reverse dippers, non-dippers also had lower daytime systolic BP and much higher night-time systolic BP than the dipper phenotype.

For the community cohort, the average daytime systolic BP of dippers was higher than that of non-dippers, by 4.4 mmHg, and higher than that of reverse dippers, by 6.8 mmHg. For the hospital cohort, the differences were 4.4 mmHg and 6.5 mmHg, respectively.

The prevalence of recorded cardiometabolic comorbidity was markedly higher in the community cohort than the hospital cohort. There are several potential contributing reasons for this. The community cohort included a much higher prevalence of patients with known hypertension (62.5% versus 33.2% in the hospital cohort); such patients are more likely to be investigated for, diagnosed with, and coded for end-stage disease from hypertension, either as part of primary prevention or when receiving a diagnostic work-up for an acute presentation. The two cohorts may be fundamentally different in terms of cardiometabolic health status. Importantly, the same pattern was seen in both cohorts, with the prevalence of cardiometabolic comorbidity rising across the spectrum of phenotypes, from dipping to extreme reverse dipping; this highlights the higher cardiovascular risk status of reverse dippers and extreme reverse dippers.

### Strengths and limitations

A large dataset (21 716 patients) was used for the analysis of in-hospital BP. The study was inclusive of adult patients aged 40–75 years presenting with all medical problems, and only excluded those admitted to maternity or intensive care units. Although much smaller, the size of the community dataset was also sizeable, with 585 participants contributing ABPM data for analysis.

A markedly different prevalence of the reverse-dipping phenotype was observed between the hospital and community cohorts. Several potential factors may contribute to this and limit comparisons between the two datasets. The prevalence of diagnosed hypertension was much higher in the community cohort than the hospital cohort. In addition, variation in the number of BP measurements contributing to the 24-hour systolic BP analysis may have had an impact on the comparability of the results between the hospital and community cohorts although the median number of BP measurements per participant for the two cohorts was very similar (27 in hospital versus 28 in the community), the interquartile range was much higher in the hospital cohort, indicating greater variability in the number of systolic BP measurements available.

The aim of this study was not to compare ‘office’ or in-hospital BP with community ABPM in individuals; rather, the authors sought to analyse data for two cohorts on a population level, to determine whether the same phenotypes exist in hospital and community cohorts. Significant differences were found between the average systolic BPs of males and females in the daytime or the night-time, but this may be because of the choice of age group (40–75 years). In the authors’ previous work,^12^ it was shown that females aged <60 years had lower systolic BPs than males, but the opposite was true at age >60 years; these two phenomena would then average out in a combined age group of 40–75 years. This study used systolic BP only to compute the 24-hour BP phenotypes of the included participants, conforming to common practice in this field.^16^^–^18

### Comparison with existing literature

The review by Cuspidi *et al* gives a prevalence for reverse dipping of 3%–39%.^1^ Reverse dipping is primarily associated with obstructive sleep apnoea and arousal;^19^ measuring vital signs at night in hospital is likely to cause an arousal to wakefulness, so the 48.9% prevalence in the hospital cohort of the study presented here is very likely to be an upper bound. The prevalence of non-dipping reported here for the hospital cohort should, therefore, be interpreted within this context.

A study of patients with hypertension in a primary care setting in Europe^1^ reported a similar prevalence of reverse dipping (12.1%) to that found in the community ABPM dataset in the present study. In a substudy of 374 patients in Belgium, of whom 32.6% had been prescribed antihypertensives, reverse dipping was observed in 14.4% of patients;^20^ the figure of 10.8% for reverse-dippers in the community cohort in the present study is in line with these data, but is probably a lower bound.

Poor reproducibility of the dipper BP phenotype in individuals has previously been reported; a meta-analysis of 14 studies revealed that up to 32% of participants were inconsistent dippers (that is, dippers became non-dippers or vice versa) on repeat ABPM.^21^ It is possible, therefore, that, if this study were repeated, some patients may be categorised differently; however, this would be unlikely to affect the proportion of patients categorised into each of the four phenotypes, as a further meta-analysis of 11 relevant studies revealed no difference in the rate of systolic nocturnal BP dipping between a first and second ABPM.^21^ Furthermore, for the hospital cohort in the study presented here, the 24-hour BP profile was computed using systolic BP measurements taken throughout their hospital admission and, as such, patients in this cohort had a BP profile consisting of measurements taken from multiple days; their 24-hour systolic BP profile represented a longitudinal, averaged picture.

### Implications for research and practice

BP is measured in general practice during daytime hours when the BP of reverse dippers and extreme reverse dippers is lowest *,* thus placing them at risk of undiagnosed, or masked, hypertension. Clinicians measuring an individual’s BP may not know that the population average night-time systolic BP of reverse dippers is 8 mmHg higher than their average daytime systolic BP. Conversely, dippers experience their highest systolic BP during the time when it is measured in general practice and, hence, are more likely to be diagnosed. Therefore, 24-hour ABPMs need to be performed in order to identify those with hypertension and reverse-dipping blood pressure phenotypes. The prevalence of the non-dipping and reverse-dipping systolic BP phenotypes in both cohorts highlights the importance of measuring BP over 24 hours to detect and diagnose hypertension.

European and international guidelines for the management of hypertension include diagnostic thresholds for night-time hypertension.^22^^,^^23^ However, when ABPM is performed in the UK, the National Institute for Health and Care Excellence recommends using only daytime BP measurements to assess for hypertension.^24^ As such, not only is hypertension likely to go undetected in reverse dippers when assessed with daytime clinic BP measurements, but their elevated nocturnal measurements on ABPM, when it is performed, are likely to be disregarded; thus, there will be patients who have already received ABPM who could readily be identified as having a reverse-dipping phenotype or nocturnal hypertension simply by reviewing existing ABPM results.

The average night-time systolic BP of reverse dippers in the hospital and community cohorts of the study presented here were 132.1 mmHg and 137.7 mmHg, respectively; this was 18.0 mmHg and 24.9 mmHg higher than the average night-time systolic BP of dippers in each respective cohort. The higher average night-time systolic BP of reverse dippers in both cohorts of the study presented here is clinically important when considering cardiovascular risk and the need to treat. In most cases, when a clinical decision about whether to treat someone for hypertension is made, consideration of the 24-hour systolic BP phenotype of dipper or non-dipper is less likely to influence the decision than knowledge of the average daytime, night-time, or 24-hour BP values. This study has demonstrated that 24-hour systolic BP should be assessed in order to detect those who are non-dippers or reverse dippers with isolated nocturnal hypertension but have normal daytime clinic BP. Paradoxically, it is those individuals with the dipping systolic BP phenotype who have their highest systolic BP during the daytime period and are, therefore, more likely to be diagnosed in clinic as having hypertension. It should also be noted that people from Black and Asian populations may be differentially affected by this issue; the non-dipping phenotype is more prevalent among Black^25^ and Asian^3^ populations than White populations

In the original analysis of the hospital cohort, it was shown that peak nocturnal systolic BP increased after the age of 60 years,^12^ and previous work has shown that reverse dipping increases with age.^26^ These findings, together with the evidence presented here, demonstrate that night-time BP recorded via ABPM should form part of the clinical assessment for hypertension in the UK, as is currently recommended in Europe,^22^ and that this is particularly important for those aged ≥60 years.

Furthermore, the prevalence of reverse dipping in hospital patients, even if it is an upper bound, indicates that those without a previous diagnosis of hypertension could benefit from automatic screening of their in-hospital 24-hour systolic BP to identify whether they should receive post-discharge ABPM in the community.^27^

If the delivery of ABPM at scale is too great a burden for primary care, it is time to investigate alternative technologies for 24-hour BP monitoring^8^^,^^28^^,^^29^ in the home in an accessible and sustainable way to ensure those with night-time hypertension and reverse-dipping phenotypes do not remain undiagnosed. Future work could include an analysis of whether 24-hour diastolic BP profiles provide independent information.
